# Supplementary material for: Modeling glioblastoma heterogeneity as a dynamic network of cell states
Source: Mol Syst Biol. 2021 Sep 16;17(9):e10105. doi: 10.15252/msb.202010105 (PMC8444284; doi:10.15252/msb.202010105)
Supplement: Supplementary file 5 — Source Data for Figure 3 [file MSB-17-e10105-s001.zip › Figure3A_sourcedata/GSEA_3065/nosigdb_state6.GseaPreranked.1622718443993/NB_KI_CLUSTER5.html]

Details for gene set NB\_KI\_CLUSTER5[GSEA]

|  || Dataset | state6 |
| Phenotype | NoPhenotypeAvailable |
| Upregulated in class | na\_neg |
| GeneSet | NB\_KI\_CLUSTER5 |
| Enrichment Score (ES) | -0.6015667 |
| Normalized Enrichment Score (NES) | -2.3489246 |
| Nominal p-value | 0.0 |
| FDR q-value | 0.0 |
| FWER p-Value | 0.0 |
Table: GSEA Results Summary

  

Fig 1: Enrichment plot: NB\_KI\_CLUSTER5      
 Profile of the Running ES Score & Positions of GeneSet Members on the Rank Ordered List

  

| PROBE | GENE SYMBOL | GENE\_TITLE | RANK IN GENE LIST | RANK METRIC SCORE | RUNNING ES | CORE ENRICHMENT || 1 | HMGN2 |  |  | 679 | 0.108 | -0.0641 | No |
| 2 | HIST1H1C |  |  | 768 | 0.101 | -0.0612 | No |
| 3 | H2AFV |  |  | 782 | 0.100 | -0.0498 | No |
| 4 | EZH2 |  |  | 940 | 0.089 | -0.0563 | No |
| 5 | NUCKS1 |  |  | 1066 | 0.082 | -0.0601 | No |
| 6 | DEK |  |  | 1535 | 0.063 | -0.1057 | No |
| 7 | RHEB |  |  | 1873 | 0.051 | -0.1378 | No |
| 8 | TUBA1B |  |  | 2087 | 0.045 | -0.1564 | No |
| 9 | PTTG1 |  |  | 2304 | 0.038 | -0.1762 | No |
| 10 | ANP32E |  |  | 2570 | 0.032 | -0.2026 | No |
| 11 | MZT1 |  |  | 2946 | 0.023 | -0.2426 | No |
| 12 | CKS1B |  |  | 2985 | 0.022 | -0.2441 | No |
| 13 | PLK1 |  |  | 3116 | 0.020 | -0.2565 | No |
| 14 | RACGAP1 |  |  | 3432 | 0.012 | -0.2911 | No |
| 15 | DUT |  |  | 3596 | 0.009 | -0.3086 | No |
| 16 | HIST1H1E |  |  | 3635 | 0.008 | -0.3119 | No |
| 17 | CDC25B |  |  | 3887 | 0.003 | -0.3403 | No |
| 18 | CENPW |  |  | 3936 | 0.002 | -0.3456 | No |
| 19 | MCM7 |  |  | 4435 | -0.008 | -0.4018 | No |
| 20 | ZWILCH |  |  | 4527 | -0.009 | -0.4111 | No |
| 21 | CDKN2D |  |  | 4667 | -0.012 | -0.4255 | No |
| 22 | ANP32B |  |  | 4891 | -0.016 | -0.4490 | No |
| 23 | TEX30 |  |  | 4923 | -0.017 | -0.4504 | No |
| 24 | CDT1 |  |  | 4990 | -0.018 | -0.4556 | No |
| 25 | CENPM |  |  | 5089 | -0.020 | -0.4643 | No |
| 26 | ECT2 |  |  | 5560 | -0.030 | -0.5144 | No |
| 27 | BUB3 |  |  | 5807 | -0.034 | -0.5382 | No |
| 28 | DDX11 |  |  | 5953 | -0.037 | -0.5500 | No |
| 29 | NUDT1 |  |  | 6071 | -0.040 | -0.5584 | No |
| 30 | H2AFZ |  |  | 6079 | -0.040 | -0.5540 | No |
| 31 | CEP152 |  |  | 6298 | -0.044 | -0.5733 | No |
| 32 | RANBP1 |  |  | 6309 | -0.045 | -0.5687 | No |
| 33 | PCLAF |  |  | 6365 | -0.046 | -0.5690 | No |
| 34 | GINS2 |  |  | 6461 | -0.049 | -0.5736 | No |
| 35 | SPDL1 |  |  | 6573 | -0.052 | -0.5796 | No |
| 36 | MCM5 |  |  | 6765 | -0.058 | -0.5941 | Yes |
| 37 | MXD3 |  |  | 6808 | -0.059 | -0.5913 | Yes |
| 38 | TCF19 |  |  | 6859 | -0.061 | -0.5892 | Yes |
| 39 | CIP2A |  |  | 6905 | -0.062 | -0.5864 | Yes |
| 40 | PRC1 |  |  | 6918 | -0.062 | -0.5797 | Yes |
| 41 | HIST2H2AC |  |  | 6963 | -0.064 | -0.5766 | Yes |
| 42 | BIRC5 |  |  | 7024 | -0.066 | -0.5750 | Yes |
| 43 | SHCBP1 |  |  | 7076 | -0.067 | -0.5722 | Yes |
| 44 | HMGB1 |  |  | 7186 | -0.071 | -0.5756 | Yes |
| 45 | RAN |  |  | 7221 | -0.072 | -0.5703 | Yes |
| 46 | MIS18BP1 |  |  | 7253 | -0.073 | -0.5645 | Yes |
| 47 | TRIP13 |  |  | 7291 | -0.074 | -0.5591 | Yes |
| 48 | ZWINT |  |  | 7370 | -0.077 | -0.5582 | Yes |
| 49 | MCM3 |  |  | 7597 | -0.086 | -0.5730 | Yes |
| 50 | SGO2 |  |  | 7632 | -0.087 | -0.5657 | Yes |
| 51 | DBF4 |  |  | 7639 | -0.088 | -0.5551 | Yes |
| 52 | HIST1H4C |  |  | 7641 | -0.088 | -0.5439 | Yes |
| 53 | GMNN |  |  | 7651 | -0.088 | -0.5335 | Yes |
| 54 | UBE2S |  |  | 7818 | -0.097 | -0.5401 | Yes |
| 55 | CHEK1 |  |  | 7852 | -0.099 | -0.5312 | Yes |
| 56 | CHAF1A |  |  | 7967 | -0.105 | -0.5307 | Yes |
| 57 | LIG1 |  |  | 7987 | -0.106 | -0.5192 | Yes |
| 58 | KPNA2 |  |  | 8045 | -0.110 | -0.5116 | Yes |
| 59 | PCNA |  |  | 8061 | -0.111 | -0.4990 | Yes |
| 60 | CKS2 |  |  | 8077 | -0.112 | -0.4863 | Yes |
| 61 | LBR |  |  | 8242 | -0.125 | -0.4889 | Yes |
| 62 | FBXO5 |  |  | 8310 | -0.133 | -0.4795 | Yes |
| 63 | DIAPH3 |  |  | 8315 | -0.134 | -0.4626 | Yes |
| 64 | NASP |  |  | 8318 | -0.134 | -0.4456 | Yes |
| 65 | CKAP2 |  |  | 8330 | -0.136 | -0.4293 | Yes |
| 66 | CDCA7 |  |  | 8337 | -0.137 | -0.4124 | Yes |
| 67 | CDK1 |  |  | 8351 | -0.139 | -0.3959 | Yes |
| 68 | ATAD2 |  |  | 8359 | -0.141 | -0.3786 | Yes |
| 69 | NCAPD2 |  |  | 8367 | -0.142 | -0.3611 | Yes |
| 70 | FANCA |  |  | 8384 | -0.144 | -0.3443 | Yes |
| 71 | HMGB2 |  |  | 8388 | -0.145 | -0.3259 | Yes |
| 72 | CLSPN |  |  | 8440 | -0.151 | -0.3123 | Yes |
| 73 | UBE2T |  |  | 8489 | -0.159 | -0.2973 | Yes |
| 74 | TUBB4B |  |  | 8554 | -0.173 | -0.2823 | Yes |
| 75 | KIF20B |  |  | 8576 | -0.180 | -0.2614 | Yes |
| 76 | PRR11 |  |  | 8580 | -0.182 | -0.2384 | Yes |
| 77 | DNMT1 |  |  | 8582 | -0.184 | -0.2148 | Yes |
| 78 | DHFR |  |  | 8590 | -0.186 | -0.1916 | Yes |
| 79 | ARL6IP1 |  |  | 8628 | -0.203 | -0.1697 | Yes |
| 80 | SMC2 |  |  | 8658 | -0.216 | -0.1452 | Yes |
| 81 | BRCA1 |  |  | 8659 | -0.217 | -0.1172 | Yes |
| 82 | CENPK |  |  | 8666 | -0.220 | -0.0895 | Yes |
| 83 | GGH |  |  | 8702 | -0.239 | -0.0626 | Yes |
| 84 | HELLS |  |  | 8720 | -0.260 | -0.0310 | Yes |
| 85 | SMC4 |  |  | 8764 | -0.307 | 0.0037 | Yes |
Table: GSEA details [plain text format]

  

Fig 2: NB\_KI\_CLUSTER5: Random ES distribution      
 Gene set null distribution of ES for **NB\_KI\_CLUSTER5**
